# Recent Surge Behavior of Walsh Glacier Revealed by Remote Sensing Data

**DOI:** 10.3390/s20030716

**Published:** 2020-01-28

**Authors:** Xiyou Fu, Jianmin Zhou

**Affiliations:** 1 Institute of Remote Sensing and Digital Earth, Chinese Academy of Sciences, Beijing 100094, China; fuxy@radi.ac.cn; 2 College of Resoures and Environment, University of Chinese Academy of Sciences, Beijing 100049, China

**Keywords:** Walsh Glacier, surge behavior, remote sensing, glacier velocity, surface elevation changes

## Abstract

Many surge-type glaciers are present on the St. Elias Mountains, but a detailed study on the surge behavior of the glaciers is still missing. In this study, we used remote sensing data to reveal detailed glacier surge behavior, focusing on the recent surge at Walsh Glacier, which was reported to have surged once in the 1960s. Glacial velocities were derived using a cross-correlation algorithm, and changes in the medial moraines were interpreted based on Landsat images. The digital elevation model (DEM) difference method was applied to Advanced Spaceborne Thermal Emission and Reflection Radiometer (ASTER) DEMs to evaluate the surface elevation of the glacier. The results showed that the surge initiated near the conjunction of the eastern and northern branches, and then quickly spread downward. The surge period was almost three years, with an active phase of less than two years. The advancing speed of the surge front was much large than the maximum ice velocity of ≈14 m/d observed during the active phase. Summer speed-ups and a winter speed-up in ice velocity were observed from velocity data, with the speed-ups being more obvious during the active phase. Changes in the glacier velocity and the medial moraines suggested that the eastern branch was more affected by the surge. The DEM differencing results showed that the receiving zone thickened up to about 140 m, and the upstream reservoir zone became thinner. These surge behaviors, as characterized by remote sensing data, gave us more detailed insights into the surge dynamics of Walsh Glacier.

## 1. Introduction

Surge-type glaciers experience well-defined, cyclical non-steady flow, alternating between a short active phase (months to years), usually characterized by rapid terminus advance, and a longer quiescent phase (years to decades), characterized by terminus stagnation or retreat [1,2]. During the active phase of a typical glacial surge, a large portion of the ice mass is rapidly transported from a reservoir area to a receiving area, leading to dramatic changes in glacial surface elevation and sometimes terminus advance. The shear stress near the glacier side margins increases during the surge, which makes crevasses more developed [3]. Furthermore, rapidly forming looped-moraines can be observed on the glacier. In the quiescent phase after the surge, ice flow slows or becomes stagnant in the lower part, leading to ice mass accumulation upstream, and retreat and thinning in the lower stream.

Due to the significant volume of ice mass that is transported rapidly downstream during the surge period, glacial surges may cause disasters. For example, in 2002, the Kolka Glacier catastrophe caused more than 100 deaths in the Caucasus [4]. The surges of Hubbard in 1986 and 2002 induced the largest glacial-lake outburst flood events ever recorded in history [5]. In Western China, the surge-induced avalanche of Karayaylak Glacier destroyed more than 1000 ha of grazing meadow and over 61 herdsmen’s houses [6]. Thus, the identification and study of glacier surge could help to prevent property damage and protect human life. Meanwhile, as glacier mass balance is usually estimated by assuming glaciers are only nonsurge-types, where the dynamics only change in response to long-term climate change, the identification of surge-type glaciers is important to better simulate future ice dynamics [7]. Although glacier surging has been recognized by scientists since the earliest study of Alaskan glacial surges during the 1890s, the mechanics of surging remain poorly known [8,9], due to the difficulty of in situ observations leading to a limited number of detailed observations being performed over a complete surge-cycle.

Recent advances in remote sensing techniques allow for large-scale movement over short periods of time. The characteristic changes in surface velocity, thickness, terminus, and surface features make it possible to identify and study surge-type glaciers using remote sensing techniques. Thus, glacier surge dynamics have been studied in many areas using remote sensing data [3,10,11,12,13,14,15,16,17], with some important progress being made regarding our understanding of glacial surge mechanisms [18,19,20]. Although Walsh Glacier was reported to surge in the 1960s, the details of the surge behavior are limited, which may partly be due to the shortage of satellite images at that time. The free distribution, long historical archives, frequent repeat cycles, and high spatial resolution of Landsat images make them excellent resources to detect surface changes of the Earth over periods of weeks to decades [21]. In particular, with 12 bit radiometric quantization, compared to the eight bits per channel used in earlier instruments, Landsat 8 Operation Land Imager (OLI) panchromatic band imagery possesses a signal-to-noise ratio nearly an entire order of magnitude better than Landsat 7 Enhanced Thematic Mapper (ETM) and previous sensors, with much less saturation for bright targets [22]. These features, accompanied by higher geolocation accuracy by nearly half a pixel and high spatiotemporal data acquisition rates of 16 days, enable us to monitor glacier changes in large quantities and high quality [23].

In this paper, we used remote sensing data to reveal recent detailed surge behaviors of Walsh Glacier, focusing on changes in glacier velocities, medial moraines, and the surface elevation of the glacier. Glacier velocities were derived using a cross-correlation algorithm to reveal the spatial and temporal changes in ice velocity associated with the surge. We interpreted the changes in the medial moraines from Landsat images, and used four Advanced Spaceborne Thermal Emission and Reflection Radiometer (ASTER) digital elevation models (DEMs) to evaluate the surface elevation changes of the glacier to better characterize the surge dynamics over the surging period.

## 2. Study Area and Datasets

### 2.1. Study Area

**Figure 1:**
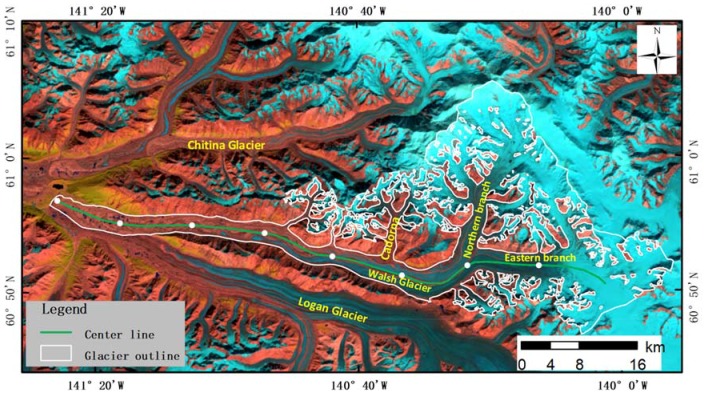
Landsat 8 image (bands 6, 5 and 4 combined) of Walsh Glacier on 11 August 2013, superimposed with the Glacier boundary and manually delineated centerline. The white dots mark 10 km intervals from the terminus.

Walsh Glacier is located in the St. Elias Mountains (Figure 1), which straddle the border between Alaska, Yukon, and British Columbia. With a glaciated area of 33,170 km^2^, the St. Elias Mountains make up one of the largest extra-polar ice masses in the world [24]. There are numerous surge-type glaciers in this area [25], but only a few have been studied in detail in the literature [3,12,13,26].

Walsh Glacier is 90.5 km in length and 718 km^2^ in coverage [24]. It flows westward from an elevation of 5241 m a.s.l. and terminates at 792 m a.s.l. It is mainly fed by two major branches, one from the north and one from the east, each ≈27 km long. Below the juncture of the two branches, the glacier trends into Alaska in the western direction and joins the Logan Glacier above the latter’s terminus at an elevation of 1130 m a.s.l. There are also three tributaries downstream of the glacier, named the first tributary, the second tributary, and the Cadorna Glacier, which are located at nearly 30, 40 and 50 km from the terminus, respectively. Walsh Glacier was reported to surge very rapidly in late 1960 or 1961 after 40 years or more of visual stagnation in the terminal area [27]. The displacement of surface features estimated from aerial photographs showed that peak movement occurred in the central portion of the glacier between 1961 and 1965, with a maximum magnitude of 10.1 km [27].

### 2.2. DataSets

Landsat 8 OLI images from 2013 to 2018 were used to estimate the surface speed evolution of the glacier and analyze the change in the medial moraines (Table 1). Launched on 11 February 2013, the Landsat 8 satellite was equipped with the Operational Land Imager payload, which was designed for moderate resolution imaging in the visible, near-infrared (NIR), and short-wave infrared (SWIR) spectral bands [28]. The repeat cycle of the Landsat 8 image is 16 days, and the scene coverage is 185 × 170 km. Furthermore, compared with previous Landsat sensors, there were some enhancements of the Landsat 8 OLI sensor, such as improved geometric accuracy, reduced noise, a larger dynamic range, reliable calibration, and increased spectral information content [21,29]. Thus, the Landsat 8 images are a potential source of remotely sensed data to monitor mountain glacier dynamics. We used images from band 8 with 15 m resolution to derive the glacial velocities. All of the images used in this study are in the same path/row (63/17) to minimize geometric distortion. The images were L1T products which were gathered, with systematic radiometric and terrain corrections, using ground control points (GCPs) and DEMs, with a geometric accuracy of within 13 m [29]. All of the images are freely distributed by the United States Geological Survey (USGS) EarthExplorer (http://earthexplorer.usgs.gov).

Four ASTER DEMs were used to derive elevation changes of the glacier during the surge (Table 1). The ASTER sensor is an imaging instrument onboard the Terra satellite platform, which is a multi-national scientific satellite equipped with multiple high-performance sensors. Although it was launched in 1999, the satellite is still in use. The ASTER captures high-spatial-resolution data in 14 bands from visible to thermal infrared wavelengths, and also has a stereo-viewing capability to create a digital elevation model with a spatial resolution of 15 m [30]. Four ASTER DEMs which were not affected by clouds were selected to evaluate the elevation change from 2006 to 2017. The ASTER DEMs were freely downloaded from the METI AIST Data Archive System (MADAS). The accuracies of ASTER DEMs range from 7 to 20 m [31,32]. Due to the limited coverage of an ASTER DEM, the four selected DEMs only covered part of the Walsh Glacier.

We also used outlines of the glacier available from the Randolph Glacier Inventory (RGI) version 5.0 [24] to subset the glacier velocity and DEM difference results, to determine regions of stable ground to assess uncertainty, and to visually interpret the changes in the medial moraine.

## 3. Methods

### 3.1. Cross-Correlation Method to Derive Glacier Surface Speed

We used the cross-correlation algorithm implemented in the frequency domain, which is integrated into the Co-registration of Optically Sensed Images and Correlation (COSI-Corr) software package [33], to estimate the glacial surface velocities. COSI-Corr has proven to obtain accurate results in many glacier applications [26,34,35]. Orientation images were used to perform the cross-correlation algorithm in COSI-Corr because they are not affected by solar illumination or areas of uniform brightness, such as featureless snow patches in the accumulation area. Orientation images were first produced using image gradients and were then inserted into the COSI-Corr software to estimate the velocities of Walsh Glacier [26]. By performing the cross-correlation algorithm in the frequency domain, the COSI-Corr could make use of the Fourier shift theorem to estimate the shift between two images. Transforming the images into the frequency domain allows for efficient estimation of the displacement between two images. More details regarding the principle of the algorithm can be found in [33].

We set the initial search window to 128 × 128 pixels and the final window to 32 × 32 pixels, which was obtained via extensive experiments. The threshold of the signal-to-noise ratio was set to 0.95 to avoid correlation bias, and four robustness iterations were applied. We set the step size to 12 pixels to generate velocity maps with a resolution of 180 m.

The post-processing steps involved a median low-pass filter, and exclusion of miscorrelations based on a priori knowledge of flow directions from surface features, distribution, and the lateral continuity of flow speeds. First, we masked all of the displacement vectors outside the glacier outlines obtained from RGI version 5.0 [24]. The remaining vectors were then filtered using a median low-pass filter. We removed vectors if they deviated by more than two standard deviations from a median vector that was computed within 3 × 3 pixel windows [36,37]. The vector field was then visually inspected, and any remaining anomalous vectors visible were manually removed. Finally, the vector field was scaled into units of meters per day (m/d) for comparison.

As there was no obvious motion on the off-glacier areas between two imaging time periods, the mean displacements on the off-glacier areas were calculated and divided by the number of days in the interval to obtain the uncertainty of the glacier velocity [38,39,40]. In order to analyze the temporal development of surface velocities in more detail, we manually digitized a centerline along the main glacier. Profiles along the centerline were extracted from the computed velocity fields to reveal the temporal development of surface velocities associated with the surge.

### 3.2. Interpretation of the Medial Moraine Changes

During the glacier surge, the position of the medial moraine may change. Landsat 8 images (band 8) at nearly one-year intervals were used to manually delineate the medial moraine and interpret its changes associated with the glacier surge.

### 3.3. DEM Difference Method

During the surge, the ice mass may be transported downstream, leading to changes in the glacier’s surface elevation. Thus, DEM difference method can be used to identify the changes of glacier surface elevation during the surge [14,41,42,43]. To minimize the horizontal offset and obtain accurate elevation changes, DEMs obtained from different periods should be precisely co-registered. In [44], a strong relationship between horizontal shifts of two DEMs and the corresponding terrain slope and aspect values in the stable areas were defined:

(1) $$\frac{dh}{\tan(\alpha )}=a\cdot \cos(b-\psi )+c,$$

where *dh* is the individual elevation difference, *α* is the terrain slope, ψ is the terrain aspect, *a* is the magnitude of the horizontal shift, *b* is the direction of the shift vector, and *c* is the mean bias between the DEMs divided by the mean slope tangent of the terrain. The cosine parameters *a*, *b* and *c* can be obtained by solving the linear equation system using a least-square optimization. The final magnitude and direction of the shift vector are calculated from the cosine parameters. By iteratively solving the Equation (1) and shifting the slave DEM, subpixel accuracy co-registration of two DEMS can be achieved.

**Figure 2:**
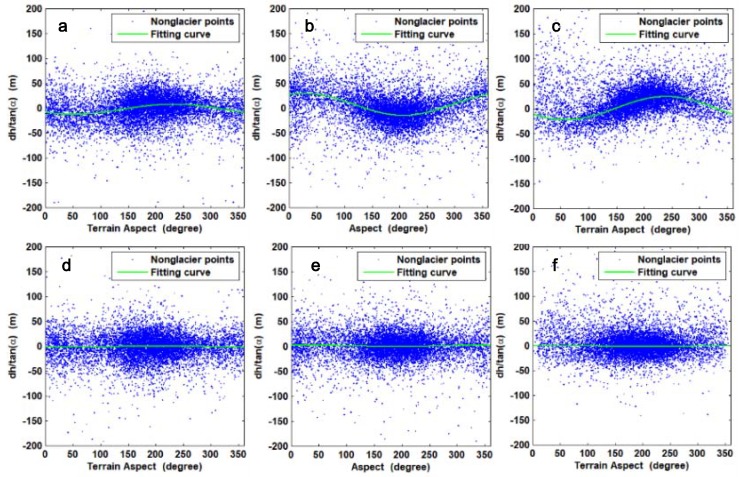
Scatterplots of slope standardized elevation differences versus aspect and fitting curve before and after co-registration. (**a**–**c**) Scatterplots for 20110830–20060401, 20150528–20110830 and 20160928–20150528 before co-registration, respectively; (**d**–**f**) scatterplots for 20110830–20060401, 20150528–20110830 and 20160928–20150528 after co-registration, respectively.

We followed the method proposed in [44] to co-register two DEMs with robustness and high precision [45,46]. In practice, pixels were eliminated by applying a threshold of ±200 m to the calculated elevation differences to avoid errors caused by clouds [46]. We excluded all pixels with a slope angle less than 5°, as these aspect values might be falsified by inherent elevation errors and we stopped the iteration when the length of the shift vector fell below 1 m [47]. The relationships between the vertical deviations normalized by the slope tangents and terrain aspects for pixels at stable regions before and after co-registration are shown in Figure 2. After performing the procedure, we estimated the mean elevation differences over stable regions before and after correction. The normalized median absolute deviation (NMAD) was also used to describe the precision of the elevation difference, as in [46], as it was proven to be a more robust statistical description than the standard deviation (SD) because the NMAD is less sensitive to outliers [48]. The NMAD is defined as [48,49]:

(2) NMAD=1.483×med(|H−med(H)|),

where *H* is a set of elevation differences in stable areas and med(·) means the median operator.

We filtered the differencing maps using a 3 × 3 low-pass filter to obtain smooth results. Some visual artifacts in the accumulation area induced by cloud, shadow, and snow-cover were removed, because the optical images had poor textures due to the saturation of images in these regions [14].

## 4. Results

### 4.1. Ice Flow Evolution between 2013 and 2018

**Figure 3:**
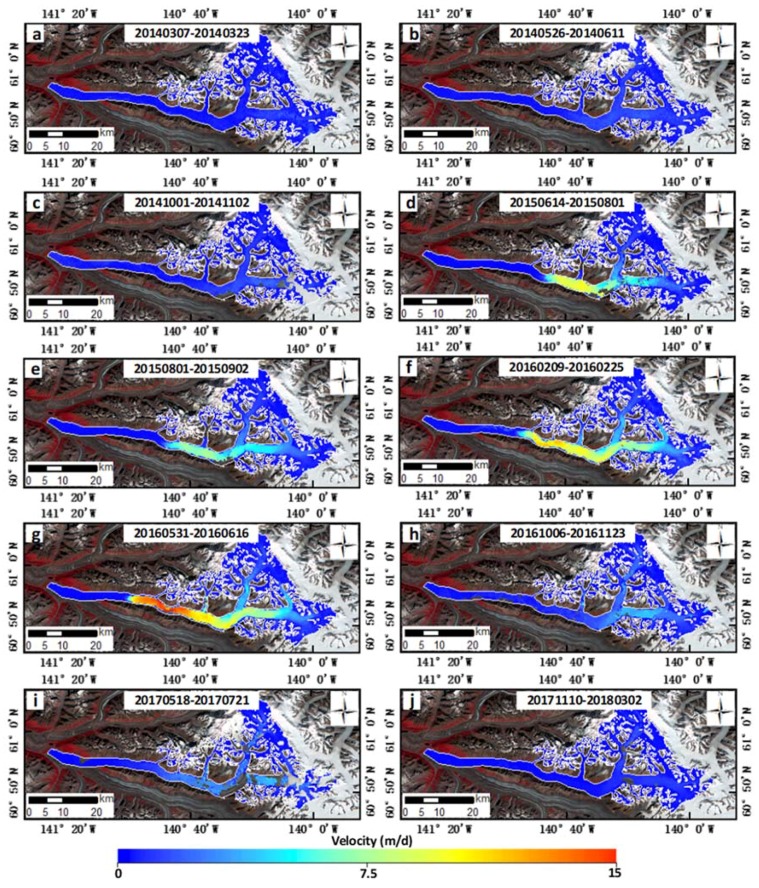
Selected velocity detail maps from March 2014 to March 2018 derived from Landsat 8 images, highlighting the seasonal fluctuations of glacier velocity associated with the surge. The velocity maps overlay the Landsat 8 image (bands 5, 4 and 3, combined) of Walsh Glacier on 11 August 2013.

We derived 46 velocity maps from 47 Landsat images associated with the surge period. Selected velocity detail maps from August 2013 to March 2018 derived from the Landsat 8 images are shown in Figure 3, highlighting the fluctuations in the glacier’s velocity. Due to the impacts of clouds, snow-cover, and shadow, there were some voids in the velocity maps. As shown in Figure 3a–c, the velocities of Walsh Glacier were low in 2014 (≈0.4 m/d). As shown in Figure 3d, the velocity accelerated to nearly 8 m/d. From Figure 3d–g, the surge spread down the glacier rapidly, with the surge front propagating downstream to almost 20 km from the terminus. The velocity of the area from the terminus to nearly 20 km from the terminus remained low during the observed time. Figure 3h,i shows that the glacier decelerated sharply to lower velocities, and Figure 3j shows the glacier in its quiescent phase. The surge at the main trunk of Walsh Glacier also triggered Cadorna Glacier to surge (Figure 3g–j). During the surge, the velocities on the eastern branch were higher than those on the northern branch.

**Figure 4:**
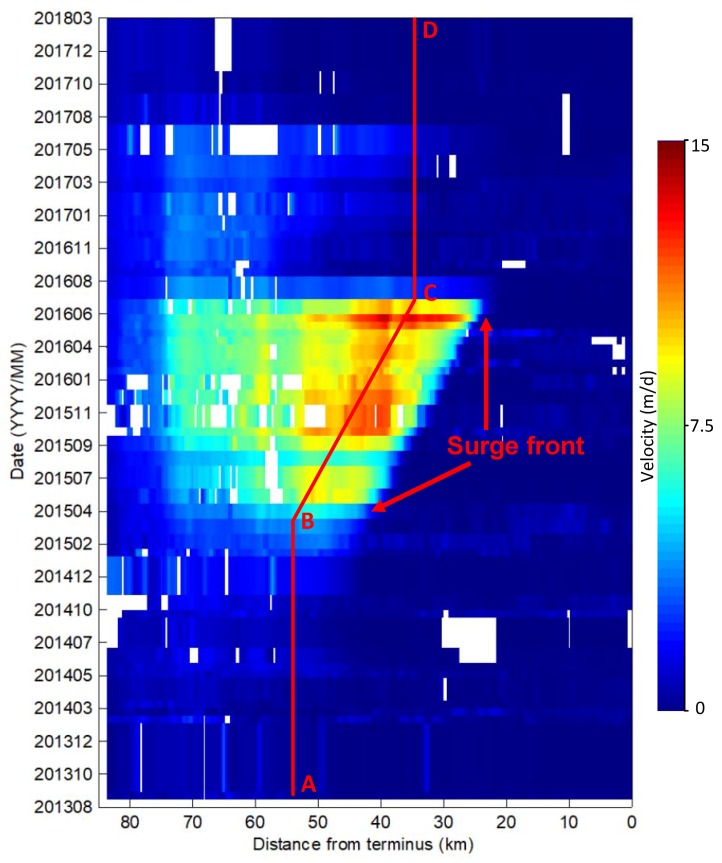
Spatial-temporal changes of surface velocities along the centerline, whose location is marked in Figure 1. The width of each profile indicates the interval of the image pair. The right edge is near the terminus and the left edge is upstream. The red line in the figure is a manually delineated line used to reveal the fluctuation of glacier velocity. A, B, C and D mark the locations of the inflection points along the line.

To better access the temporal variations of glacier velocity, we extracted the centerline profiles of the estimated surface velocity along the glacier, as the velocity along the centerline was considered to be the most accurate and robust. The time–distance plot was drawn to reveal temporal changes in the velocity profiles (Figure 4). The voids in the velocity profile are shown in white in the time–distance plot. It can be seen that, before winter 2014, the velocity below 50 km from the terminus remained very low. From winter 2014, the velocity accelerated at 50–60 km from the terminus, which was almost where the conjunction of the eastern branch and northern branch was situated, as seen from Figure 1. The surge then rapidly spread downward. The advance of the surge front was easily recognized. The surge front moved toward the terminus of the glacier very quickly at a moving distance of ≈20 km in less than two years, from winter 2014 to summer 2016. The surge front did not propagate to below 20 km from the terminus, as the velocity dropped sharply in the summer of 2016. From summer 2016, a mini-surge, which ended in summer 2017, was observed between 50 and 70 km on the eastern branch. After summer 2017, the glacier turned quiescent, with the velocity reduced to a very small magnitude.

**Figure 5:**
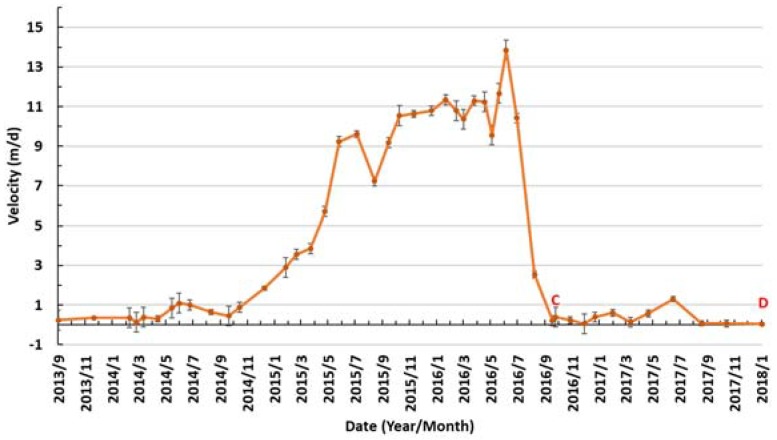
Temporal changes in the glacial surface velocity along the red line, as shown in Figure 4. The error bars indicate the uncertainty in the velocity measurements.

The velocity profile, marked by the red line in Figure 4, was extracted to examine the detailed temporal changes in ice speed (Figure 5). Summer speed-ups in the glacier in summer were clearly observed. The glacial velocity between 2013 and 2014 was almost stable at ≈0.5 m/d (Figure 5), but there was a small seasonal speed-up in the summer. From winter 2014, the velocity continued to accelerate rapidly until it reached more than 9 m/d in the summer of 2015. After a drop in the late summer of 2015, the velocity continued to be high, although several small drops were experienced. After reaching the peak velocity of ≈14 m/d, the velocity dropped sharply to less than 1 m/d in the late summer of 2016. A summer speed-up was seen in the summer of 2017. From autumn 2017, the velocity dropped to a very low magnitude.

### 4.2. Changes in the Medial Moraine

**Figure 6:**
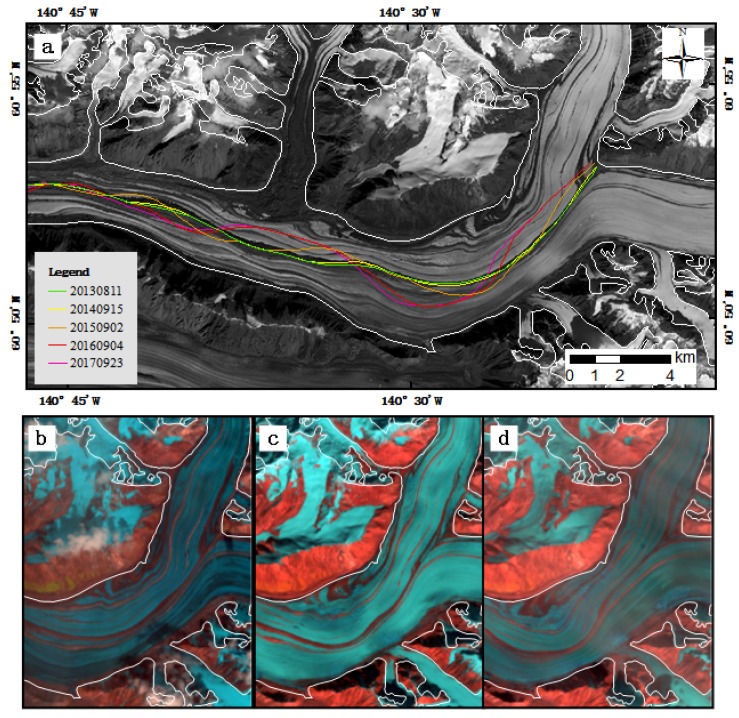
Evolution of the medial moraine associated with the surges. (**a**) Manually delineated medial moraines overlaid on Landsat images (band 8) from 11 August 2013. (**b**–**d**) Landsat images (bands 6, 5 and 4, combined) from 15 September 2014, 2 September 2015 and 4 September 2016, respectively.

We manually delineated the medial moraine from false-color composite Landsat images from 2013 to 2017 to clearly show the evolution of the medial moraine associated with the surges (Figure 6a). From 11 August 2013 to 15 September 2014, there was no obvious change in the medial moraine. From 15 September 2014 to 2 September 2015, as the surge initialized and spread downward, the ice speed of Walsh Glacier accelerated. The surge speed at the eastern branch was greater than the northern branch, thereby pushing the medial moraine at the upper portion of the conjunction toward the west, and changing the downstream position of the medial moraine as a waveform compared with that observed on 15 September 2014. From 2 September 2015, as the glacier continued to accelerate, the upper portion of the medial moraine was pushed more toward the west and the position of the medial moraine changed greatly from that observed on 2 September 2015. From 4 September 2016, the glacier decelerated substantially, with only slight changes of the medial moraine being observed. Selected images highlighting the change in the medial moraine are shown in Figure 6b–d.

### 4.3. Surface Elevation Changes

**Figure 7:**
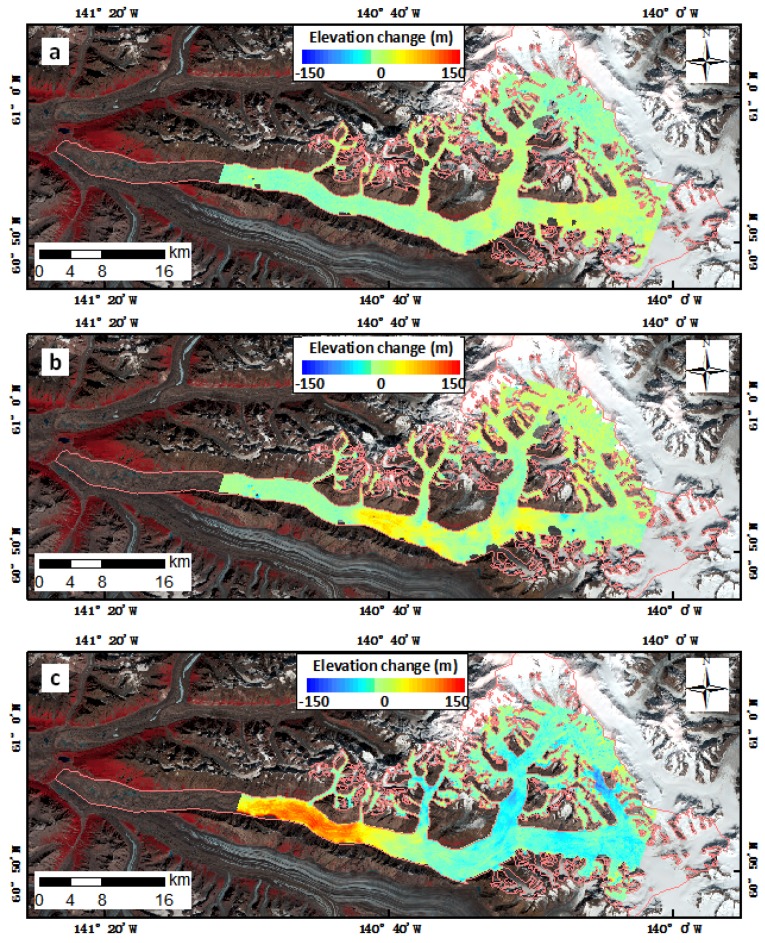
Digital elevation model (DEM) differencing results of Walsh Glacier overlain on Landsat 8 image (bands 5, 4 and 3, combined) of Walsh Glacier on 11 August 2013. (**a**) 20110830–20060401. (**b**) 20150528–20110830. (**c**) 20160928–20150528.

The spatial and temporal changes in glacial surface elevation associated with the surging event were revealed by the DEM differencing results derived from the ASTER DEMs (Figure 7). The DEM differencing results covered only part of the Walsh Glacier due to the limited coverage of the ASTER DEM, but it still revealed the surface elevation changes associated with the surge. After the surge, the downstream area of Walsh Glacier thickened, whereas the eastern branch, northern branch, and the Cadorna Glacier became thinner (Figure 7). The surge initiated from the main trunk of Walsh Glacier and triggered the surge of the eastern and northern branches and Cadorna Glacier, leading to a rapid loss of ice mass in these areas, as validated by the velocity data.

**Figure 8:**
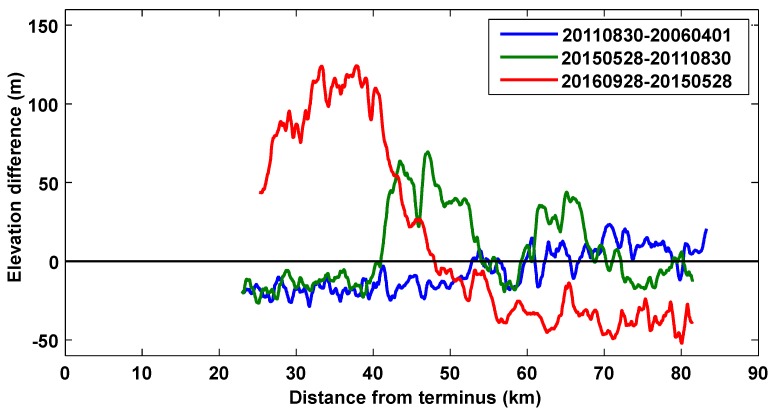
Centerline profiles extracted from the DEM differencing results.

We extracted the centerline profiles from the results of the DEM difference method, as shown in Figure 8. Between 2006 and 2011, the glacier thickened by 10–20 m in the upstream area from 70 km up from the terminus, and the lower part (between 25 and 50 km up from the terminus) of the glacier became thinner by ≈20 m (Figure 8, blue line). Between 2011 and 2015, as the surge continued, two large “bumps” in thickness occurred. The first bump, which was located on the main trunk 40–55 km up from the terminus, was formed by the ice mass from both of the branches almost 40–60 m high. The second “bump,” located at 70–80 km on the eastern trunk, was formed by the ice mass which was transported down from the top of the eastern trunk. From 2015 to 2016, the thickened regions moved downstream and merged together, further thickening at the receiving zone with a maximum of nearly 140 m, whereas the glacier thinned by 20–50 m at the reservoir zone.

## 5. Discussion

### 5.1. Uncertainty

We assessed the uncertainty of the glacial movement by measuring the average displacement in the off-glacier area. The averaged displacement in the off-glacier area was smaller than 8 m in every Landsat-derived displacement map with a period of 16–144 days, which is consistent with previous studies [38,39,40]. The uncertainty in the derived velocity was presented as error bars along with velocity measurements, as shown in Figure 5.

The NMAD is a robust statistical description equal to the standard deviation used to describe the uncertainty of elevation difference [48]. After co-registration, the mean of the stable areas improved greatly to nearly 0. The NMAD of each image pair from the stable areas was smaller than 15 m, as shown in Table 2. As the elevation change during the surge was large, with the maximum change larger than 140 m, the accuracy of the DEM differencing results was high enough to characterize the changes in the surface elevation of the glacier.

### 5.2. Comparison with the Previous Surge

We compared the surge behavior with the previous surge of Walsh Glacier. In the active phase, consisting of nearly four years from 1961–1965, the maximum movement of Walsh Glacier amounted to nearly 10 m/d, which occurred between the first tributary and Cadorna Glacier. The movement in the Cadorna Glacier vicinity was nearly 8.68 km during these periods, corresponding to a movement of nearly 8 m/d. During the active phase of the recent surge of Walsh Glacier from winter 2014/2015 to summer 2016, the mean velocity in the Cadorna Glacier vicinity was nearly 8 m/d (mean velocity shown between B and C in Figure 5), which was comparable to the velocity of the last surge in the 1960s.

From the amount of movement recorded between 1960 and 1963, Post et al. suggested that the surge started in the upper portion of the glacier in late 1960 or in 1961, and the surge completed its active cycle in about 1965, as identified from the relative absence of fresh fractures [27]. This indicates that the active phase of the last surge of Walsh Glacier was nearly four years. The active phase of the recent surge was less than two years, from winter 2014/2015 to summer 2016, shorter than that of the last surge.

During the last surge, the surface elevation of the glacier in the upper source areas was reduced by 150 m compared with its 1960 levels. In the recent surge, the maximum lowering of the eastern branch, as interpreted from the DEM differencing profile, was nearly 40 m, but the maximum lowering area was found in a tributary of the eastern branch, which thinned for ≈100 m, as shown in Figure 7c. The maximum thickening at the lower portion of Walsh Glacier was nearly 140 m.

Combined with the velocity data, the active phase, and the surface elevation changes, the magnitude of the recent surge between 2014 and 2017 was concluded to be comparable to the surge between 1961 and 1965.

### 5.3. Interpretation from Observations

As observed from the velocity data, the surge first initiated near the conjunction of the eastern and northern branches. The speed-up then rapidly spread downward, triggering surges on the eastern and northern branches. on its way downstream, the fast-flowing ice initiated a surge in cadorna glacier. the surge extent also crept upstream, and tributaries that were further up the glacier from the initial surge area started to speed up, leading to glacier-wide acceleration, except for the portion near the terminus.

The surge lasted for nearly three years, initiating in winter 2014 and ending at the end of summer 2017. the active phase of the surge was less than two years, from winter 2014/2015 to summer 2016, as seen from the ice velocity data and the changes in the medial moraines. the maximum ice velocity was apparent in the summer of 2016, with a magnitude higher than 14 m/d. then, the glacial velocity experienced a sharp decrease in velocity in the summer. summer speed-ups in ice velocity were observed from the velocity data. the speed-ups were more obvious during the active phase, suggesting the importance of seasonal meltwater input from the surface in maintaining high velocities, as basal sliding was enhanced when the surface meltwater drained through the ice and reached the bed through crevasses that were created during the active phase. these phenomena are the typical signs of an alaskan-type surge controlled by the hydrological regime [7,8,50,51]. seasonal meltwater can also be stored inside the ice body if the basal drainage becomes even more inefficient due to the rapid traveling of the velocity front during the active phase. the seasonal water stored inside the ice body is used to maintain high speed in winter even in the absence of surface meltwater [52,53], which may be the reason why a speed-up in the ice velocity occurred in winter 2015. ice velocity speed-ups in winter or spring were also observed at other surging glaciers and were attributed to being mainly induced by hydrological causes [3,10,20].

Interestingly, during the active phase, the surge front advanced nearly 20 km in less than two years, from winter 2014/2015 to summer 2016, corresponding to more than 30 m/d. this advancing speed of the surge front was much larger than the maximum velocity of ≈14 m/d during the active phase, which was also seen in other glaciers and may be related to a kinematic wave traveling downstream or some other mechanism near the glacier bed that activated the flow [38].

The glacier velocity on the northern branch was higher than that on the eastern branch during the surge, as shown in Figure 3d–g. The medial moraine near the conjunction was also pushed toward the west as the surge continued. These features suggested that the eastern branch was more affected by the surge.

Going further back in time, [27] observed that Walsh Glacier began a rapid surge in late 1960 or 1961, with the maximum movement of nearly 10 km occurring in the central portion of the glacier between 1961 and 1965, and also suggested that the glacier made an unrecorded surge sometime around 1918 [27]. As the recent surge occurred from nearly 2014–2017, we speculate that the surge cycle of Walsh Glacier is likely to be more than 40 years.

### 5.4. Advantages and Disadvantages of the Data and Methods Used

We used a cross-correlation algorithm in the frequency domain (COSI-Corr software package) to derive the glacial velocities and interpret the changes in the medial moraine from Landsat 8 images. COSI-Corr software was proven to produce velocity results with high accuracy compared with other methods [37]; thus, it is widely used to estimate glacial velocity [34,35,54,55]. The better signal-to-noise ratio and lesser saturation for bright targets, accompanied by high geolocation accuracy and spatiotemporal data acquisition rates, make Landsat 8 images one of the most important data sources to monitor glacier velocity [22,23]. The uncertainties of the glacier velocity estimated from off-glaciers suggested that the accuracy is in accordance with the recent studies of glacier velocity [38,39,40] and was sufficient to characterize the fluctuations and speed-ups of the velocity during the surge of Walsh Glacier. Different solar illuminations, shadows, clouds, and snow-cover were the main sources of potential correlation failure. Although all of the Landsat images used were in the same path/row (063/017) to minimize the influences of geometric distortions and shadows [56], there were still some voids in the velocity maps resulting from correlation failure. In addition, clouds also forced us to choose some image pairs with longer intervals, thereby lowering the temporal resolution of the glacial velocity. Sentinel-1 synthetic aperture radar data may serve as an alternative source to overcome the effects of clouds from July 2015, because all interferometric-wide swath data over land and ice masses are available thereafter.

The DEM difference method was used to measure the glacier’s elevation changes using ASTER DEMs before and during the surge. The accuracies of ASTER DEMs vary from 7 to 20 m, depending on the conditions [31,32]. By using the co-registration method of DEMs, we were able to compare DEMs with accuracy better than ≈20 m in this study, which is consistent with previous studies [57,58], thereby enabling us to monitor elevation changes during the glacial surge of Walsh Glacier. Due to the limited coverage of ASTER images, elevation differences were not presented for the snout region. Luckily, as seen from the spatial-temporal changes of the surface velocities along the centerline (Figure 4), the glacial surge front did not propagate to below 20 km from the terminus, suggesting that there was little change in glacial elevation in the areas below 20 km from the terminus. However, more details could be revealed if DEMs with greater spatial-temporal resolution and vertical accuracy were to be obtained.

## 6. Conclusions

In this paper, remote sensing data were utilized to characterize the recent surge behavior of Walsh Glacier. We derived the surface velocities of the glacier using the cross-correlation algorithm and the glacier surface elevation changes using the DEM difference method. Changes in the medial moraine were also delineated and interpreted from remote sensing images.

From the velocity data, we observed that the surge first initiated at the conjunction of the eastern and northern branches, and then spread quickly downward and triggered a surge on the eastern and northern branches, leading to glacier-wide acceleration. The glacier surged for nearly three years, with an active phase of less than two years. Summer speed-ups and a winter speed-up in ice velocity were observed from the velocity data, thereby suggesting the importance of seasonal meltwater input from the surface in modulating the velocity. These observed phenomena suggest that the surge of Walsh Glacier was a typical Alaskan-type, which normally initiates in winter and terminates abruptly in summer with a short active phase (1–3 years) and seasonal regulations in glacier velocity [7,8,50,51]. The maximum ice velocity occurred in the summer of 2016, with a magnitude greater than 14 m/d. It is worth mentioning that the maximum velocity only occurred for a very short period of time, while the speed of the surge front was averaged out over the whole duration. The glacier surface elevation changes showed that the receiving zone thickened up to about 140 m, and the upstream reservoir zone became thinner. The surge behavior suggested that the scale of this surge was comparable to the surge in the 1960s. Taking into account the previous surge, we speculate that the surge cycle of Walsh Glacier is more than 40 years. Although elevation differences were not presented for the snout region due to the limited coverage of the ASTER DEMs, these surge behaviors derived from remote sensing data provide useful insights into the surge dynamics of Walsh Glacier and will be of help to better understand glacial surges in this area.

## Figures and Tables

**Table 1:** Satellite image data used in this study.

Sensor Type	Path/Row	Date	Sensor Type	Path/Row	Date
Landsat 8 OLI	063/017	20130827	Landsat 8 OLI	063/017	20160225
	063/017	20130912		063/017	20160312
	063/017	20140203		063/017	20160413
	063/017	20140219		063/017	20160429
	063/017	20140307		063/017	20160515
	063/017	20140323		063/017	20160531
	063/017	20140510		063/017	20160616
	063/017	20140526		063/017	20160718
	063/017	20140611		063/017	20160904
	063/017	20140713		063/017	20160920
	063/017	20140915		063/017	20161006
	063/017	20141001		063/017	20161123
	063/017	20141102		063/017	20161209
	063/017	20150121		063/017	20170110
	063/017	20150206		063/017	20170227
	063/017	20150310		063/017	20170331
	063/017	20150411		063/017	20170518
	063/017	20150513		063/017	20170721
	063/017	20150614		063/017	20170923
	063/017	20150801		063/017	20171110
	063/017	20150902		063/017	20180302
	063/017	20151004	ASTER	**Image ID**	
	063/017	20151020		ASTB060401205946	20060401
	063/017	20151207		ASTB110830205358	20110830
	063/017	20160108		ASTB150528210110	20150528
	063/017	20160209		ASTB160928205437	20160928

**Table 2:** Statistics for non-glaciated areas before and after correction.

Date	Before Correction (m)		After Correction (m)	
	Mean	NMAD	Mean	NMAD
20060401–20110830	−0.0565	14.826	−0.0332	14.593
20110830–20150528	−1.651	17.7912	0.0957	13.3303
20150528–20160928	9.1914	17.7912	0.0511	12.4866
